# Identification of nitrite treated tuna fish meat via the determination of nitrous oxide by head space-gas chromatography/mass spectrometry

**DOI:** 10.12688/f1000research.19304.2

**Published:** 2019-08-12

**Authors:** Markus Niederer, Sandra Lang, Bernard Roux, Thomas Stebler, Christopher Hohl

**Affiliations:** 1 State Laboratory Basel-City, Kannenfeldstr. 2, 4056, Basel, Switzerland

**Keywords:** tuna, red colour stability, food fraud, nitrite, nitrous oxide, food additives

## Abstract

Tuna fish meat is an expensive and highly perishable sea food. Fresh meat has a bright red colour which soon turns into an unsightly brown during storage. To prolong the aspect of freshness, the red colour is stabilised or even enhanced e.g. with carbon monoxide or nitric oxide, the product of a nitrite / ascorbic acid treatment, which bind as a ligand to myoglobin. These procedures are illegal. Here we present a method for identifying tuna meat samples, which have undergone fraudulent wet salting with nitrite. The method uses headspace-gas chromatography/mass spectrometry (GC/MS) for the determination of nitrous oxide, which is formed as the final product of the two-step reduction nitrite (added agent) to nitric oxide (ligand) to nitrous oxide (target compound). Complex bound nitric oxide is set free with sulfuric acid, which also promotes the reduction to nitrous oxide. The method was validated using ^15^N labelled nitrite as well as treated and untreated reference fish samples. A survey of 13 samples taken from the Swiss market in 2019 showed that 45 % of all samples were illegally treated with nitrite.

## Introduction

**Figure 1.**
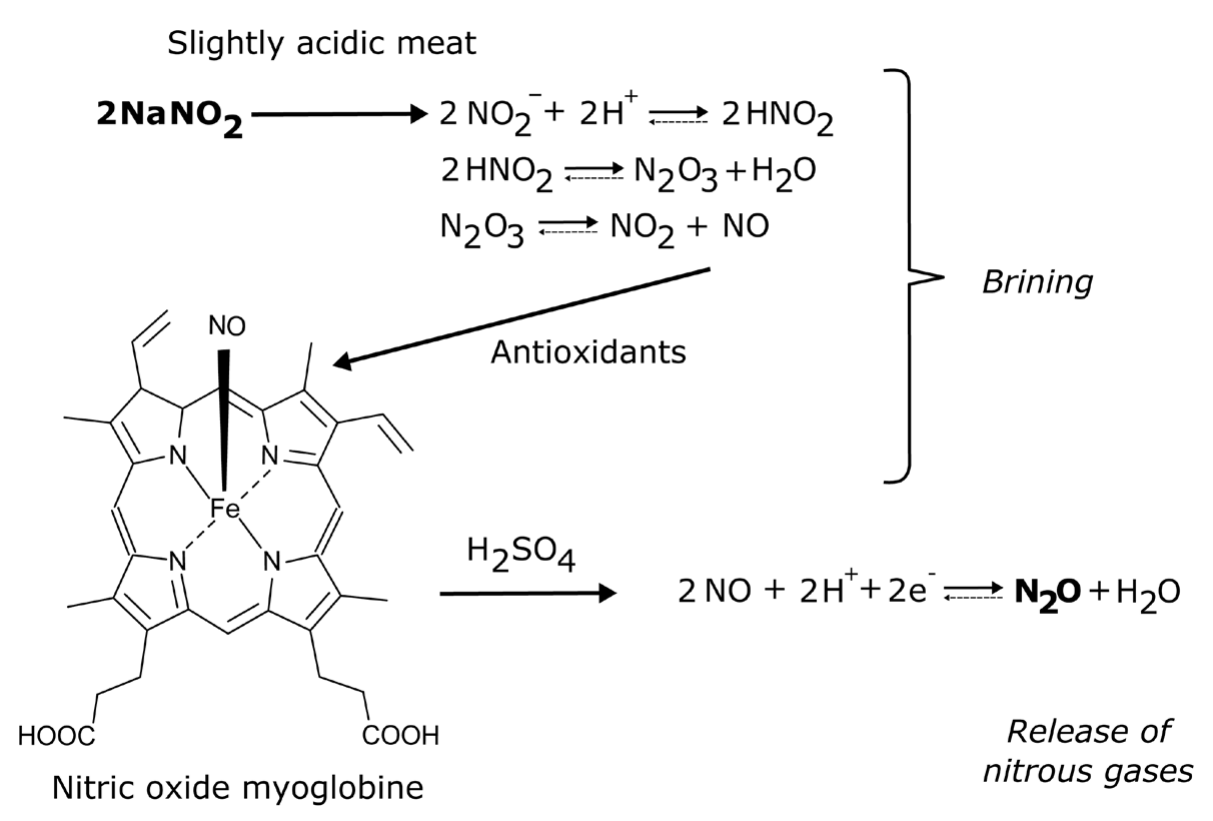
Postulated reaction schemes: brining with NO2 ^-^, formation of N _2_O as a stable reduction product of NO. In bold: verification experiment with labelled Na ^15^NO _2_ and resulting ^15^N _2_O.

As Tuna fish species have been overfished on the one hand, and their meat being a sought-after but highly perishable food stuff on the other hand, the seafood industry has felt itself constrained to undertake efforts in increasing the shelf life of this commodity. Being a bright red in its fresh state, but turning into an unsightly brown during storage, attempts have also been made in extending or even enhancing the attractive aspect of fresh meat. The red colour of meat in its natural state is due to myoglobin in its reduced form (Mb-Fe ^+II^-O _2_). Being susceptible to autoxidation, the resulting metmyoglobin (Mb-Fe ^+III^-O _2_) is brown in colour. One way of stabilising the red colour is by gassing with carbon monoxide (CO), which binds as a ligand to myoglobin (Mb-Fe ^+II^-CO). A second way consists in applying the anion nitrite (NO _2_^-^) in combination with the reducing agent ascorbic acid to form nitric oxide (NO), which forms a complex with both myoglobin (Mb- Fe ^+II^-NO) and metmyoglobin (Mb-Fe ^+III^-NO) ^1^. Whereas the colour hue of the CO complex is described as cherry red, that of the NO-complexes are described as pink red. Neither procedure is authorised for fresh fish ^2^. However, with these possibilities at hand, abuse for simulating the fresh aspect of tuna fish meat is a tempting option ^3, 4^, all the more so regarding the currently insufficient tools for fraud detection: Whereas, for CO, analytical methods are at hand to support law enforcement ^5, 6^, attempts to detect nitrite treated meat, either by searching for nitrite residues or traces of nitrosamines, have not been reported as being successful yet ^7^. Here we report on our method for the detection of nitrite treatment (brining), which originates from the method for CO determination and determines nitrous oxide (N _2_O) as the reduction product of nitric oxide (NO), stemming from the complex Mb- Fe ^+II^-NO ( Figure 1). This approach allows the determination of CO and N _2_O even within the same GC/MS run.

## Methods

### Chemicals

Sulphuric acid (95–97%, cat. no. 1.00731), 1-octanol (puriss., cat. no. 1.00991), sodium chloride (NaCl, p.a., cat. no.1.06404.1000), sodium nitrite (NaNO _2,_ p.a., cat. no. 1.06549.0100), sodium nitrite-N15 (Na ^15^NO _2, _99%, cat. no. 490814-1G), ascorbic acid (p.a., cat. no. 127) were all from Merck (Buchs, Switzerland) and sodium ascorbate (99%, cat. no. 352681000) from Acros (New Jersey, U.S.A.). Certified NO- gas (100 ppmv N _2_, 5 L, 150 bar, cat. no.1878) was purchased from Carbagas (Basel, Switzerland) and N _2_O- gas (100 ppmv N _2_, 2 L, 150 bar, cat. no. 7092325) was from Messer AG (Lenzburg, Switzerland).

### Samples and sample preparation

Tuna samples (frozen loins, tuna saku, loose and pre-prepared packages) were taken from retailers and importers in Basel and stored at – 18°C until sample preparation. An aliquot of 15 g was weighed into a 70 mL centrifuge tube and 15 g of crushed ice as well as 15 mL of water were added. The mixture was homogenised at 7,000–9,000 rpm with a polytron homogeniser (Kinematica AG, Switzerland) and centrifuged for 5 min at 3,500 rpm. An aliquot of the resulting supernatant (5 mL) was transferred into a 20 mL headspace vial. After adding 5 µL of n-octanol and 2 mL of sulphuric acid (20 % in deionised water) the vial was immediately sealed and gently shaken by hand for 30 s. Certified NO- gas 100 ppmv (122.6 ng/mL) and N _2_O- gas 100 ppmv in N _2_ (180.6 ng/mL) served as standards for a one-point calibration: A headspace vial was filled with 5 mL of deionised water and 2 mL of sulphuric acid (20 %), the cap only loosely fitted and the reference gas introduced with a needle through the septum. The cap was then sealed tight.

### Headspace – gas chromatography/mass spectrometry (GC/MS)

The sealed vials were placed into the incubation unit of the CombiPAL autosampler (CTC Analytics, Zwingen, Switzerland) for 60 min at 25°C and analysed with a Trace 1310 GC / ISQ quadrupole system (Thermo Scientific, U.S.A.) under the following conditions: 500 μL of headspace was injected at 70°C (split 1:20) onto a Porous Layer Open Tubular (PLOT) column (30 m × 0.32 mm inner diameter, 12 µm film thickness, HP-PLOT Molsieve, Agilent Technologies, cat. no. 19091P-MS4), Palo Alto, CA, U.S.A.). Helium (99.999%, Carbagas, Basel, Switzerland, cat. no. P6201RG) was used as carrier gas at a constant flow rate of 1 mL/min. The oven temperature was programmed from 70°C (5 min) to 220°C (3 min) at 30°C/min. The quadrupole detector was set in the electronic ionisation mode (full scan 10–100 amu) with selected-ion monitoring (70 eV, ion source = 170°C, delay = 2.5 min) with a transfer-line to the GC (1 m fused silica deactivated, 0.25 mm i.d.) at 200°C. Specific masses for the detection were m/z 30 for NO (m/z 31 for ^15^NO) at a retention time of about 7.9 minutes and m/z 44 for N _2_O (m/z 46 for ^15^N _2_O) at 11.4 minutes.

### Brining experiments

For brining experiments, 15 g of nine untreated fish samples (certified as 100 % tuna from the Maldives, the Philippines, China, Vietnam) were each cut into pieces of about 1 cm and treated with 20 mL of brine solution (10% NaCl and 0.4% NaNO _2_ in deionised water) ^8^ at room temperature for 24 hours. After removing the solution, the now reddish looking tuna samples along with eight untreated control samples were each bathed twice in 15 mL of antioxidant solution (3.0 % ascorbic acid and sodium ascorbate in deionised water) for one hour at room temperature and subsequently washed twice with antioxidant solution. Thereafter, samples were prepared as described above and analysed by headspace-GC/MS.

Verification experiment: The whole procedure was also done with three tuna samples using Na ^15^NO _2_ for the brine solution instead.

## Results and discussion

### Method development

In contrast to carbon monoxide, where the agent used for treatment, the ligand responsible for colour stabilisation and the target compound for sample screening is one and the same molecule, nitrite treatment identification has to consider the two-step reduction from nitrite to nitric oxide to nitrous oxide. This required changing the original GC temperature programme of the CO method: Instead of an isothermic run at 40°C, oven temperature was ramped up from 70°C to 220°C.

**Figure 2.**
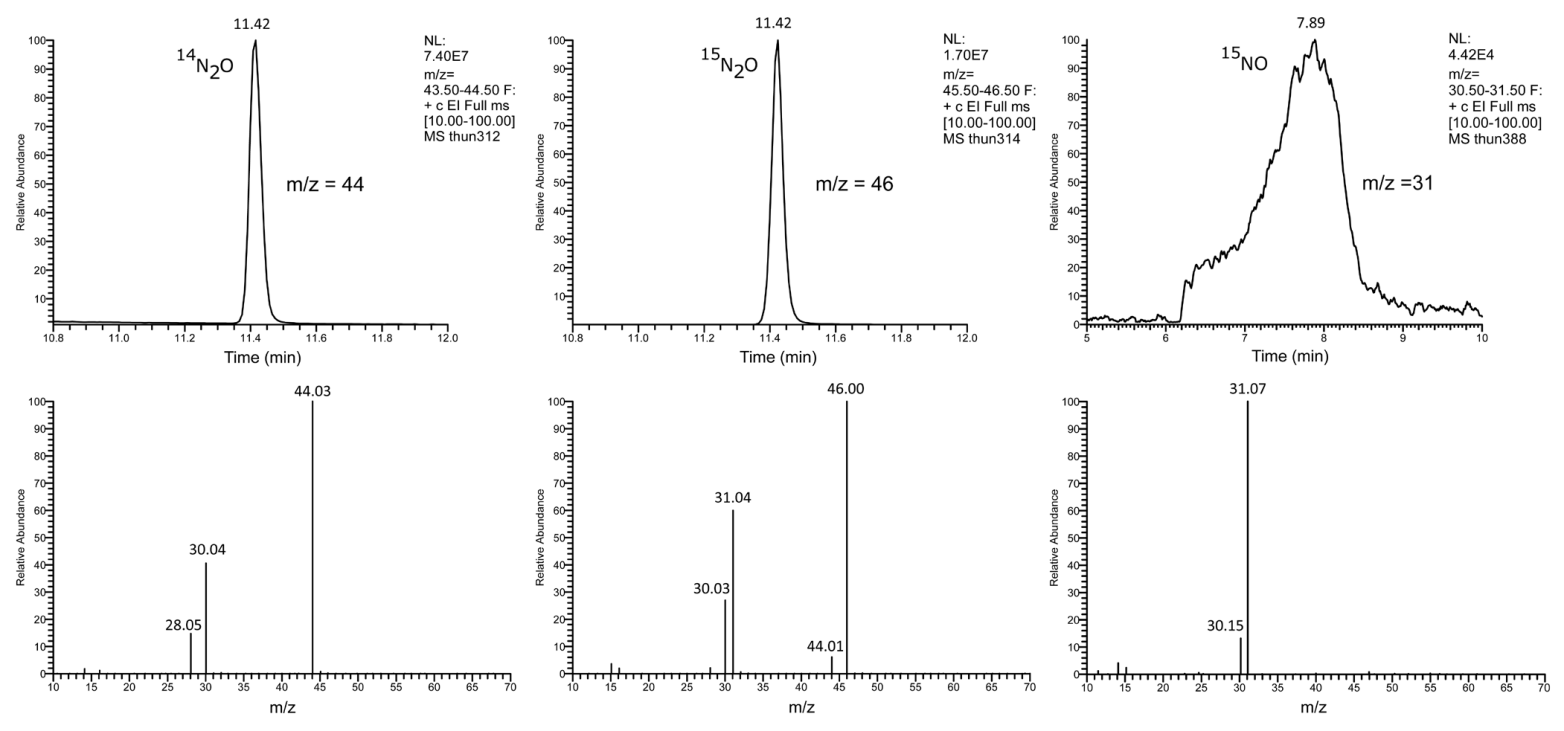
Chromatograms and mass spectra of native ^14^N _2_O as well as labelled ^15^N _2_O and ^15^NO released from nitrite treated samples.

Both carbon monoxide and nitric oxide are bound in the sample as a ligand. Their release is enhanced by adding sulfuric acid. Being a free radical, however, nitric oxide is far more reactive than carbon monoxide: Instead of a sharp symmetrical peak, chromatograms show nitric oxide as a broad hump, barely distinguishable from baseline noise. The direct approach therefore is not feasible. In an acidic environment, however, two NO molecules, upon reduction, react to nitrous oxide (N _2_O), which shows up as a perfect peak in the chromatogram ( Figure 2), allowing for the indirect detection of NO. For the purpose of identifying nitrite treatment, a semi quantitative assessment of N _2_O proved to be sufficient, as any clear signal is alone due to a former nitrite treatment.

### Brining experiments

**Figure 3.**
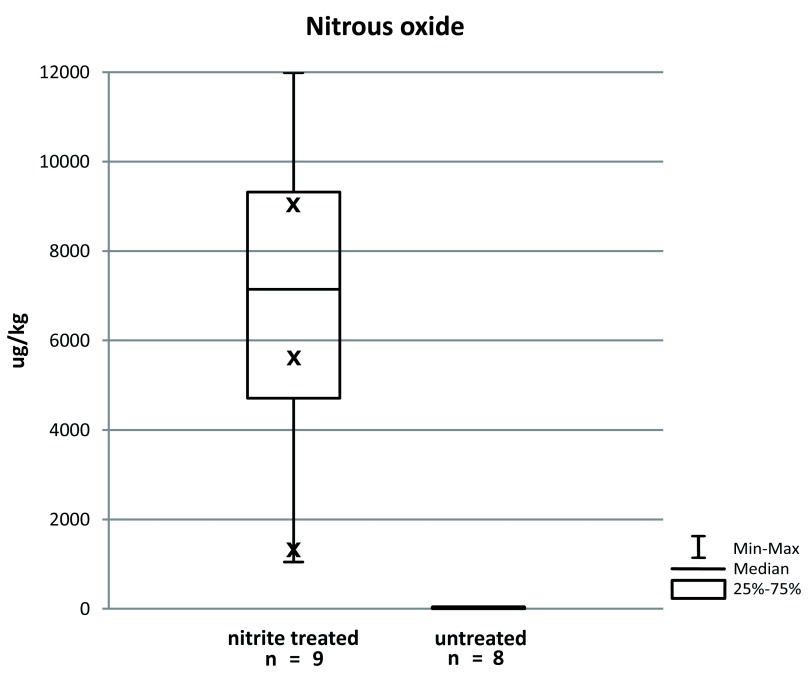
Box-and-Whisker-Plots of brining experiments. ^15^N _2_O labelled samples ( **x**).

Verification of this mechanism was performed using isotope labelled nitrite (Na ^15^NO _2_) for brining ( Figure 1). During subsequent analysis, both the ^15^NO hump and ^15^N _2_O peak were detected ( Figure 2), which is consistent with the postulated reaction pathway. In addition, brining experiments with nine tuna samples using unlabelled NaNO _2_ showed sharp N _2_O signals in the range of 1000 to 12000 μg/kg ( Figure 3, underlying data ^9^). In contrast, untreated reference samples, even though they were exposed to antioxidants, were free of nitrous oxide (limit of detection about 30 μg/kg). Therefore, naturally occurring relevant concentrations of N _2_O can be excluded.

### Validation with reference samples

The method was validated with seven reference tuna samples of a trustworthy source from 2017 which were stored at -18°C for two years in our lab (see underlying data ^10^). Five of them were declared as untreated and consists of 100% tuna. The remaining two samples were declared as treated with rosemary extract (E392) and/or nitrite and labelled with additives such as salt and antioxidants (E300, E301, E331). The untreated samples were free of N _2_O (< 30 μg/kg) whereas the treated ones showed stable N _2_O signals of 260 and 750 μg/kg. These results not only confirm our findings from the brining experiments, but are also consistent with the corresponding documents of the reference tuna samples.

### Market survey

The method was then used on a routine basis in a market survey of 13 samples of raw tuna collected in Basel in 2019 (see underlying data ^11^). Seven samples (54%) were negative. Six samples (46%) were tested positive with N _2_O peaks corresponding to levels of between 90 and 1130 μg/kg. As a consequence, these six samples, all originating from Vietnam, were objected according to law.

## Conclusion

With our experiments using labelled Na ^15^NO _2_ and the comparison of our results with the corresponding documents of the reference tuna samples, we could demonstrate that our method using nitrous oxide as a target compound is suitable for identifying nitrite manipulated tuna. Its use in a market survey also showed, that the method is fit for purpose. In addition, the method was even capable of identifying nitrite treatment in samples stored at -18°C for two years before analysis.

## Data availability

As a law enforcement body, we are bound to official secrecy. Considering this restriction, names of products and importers were anonymised.

Figshare: Fig03 brining experiment rawdata.csv. https://doi.org/10.6084/m9.figshare.8142992.v1 ^9^

This project contains the following underlying data:

- Fig03 brining experiment rawdata.csv (Raw data and descriptive statistic data (univariate diagram) of "figure 03" performed with the Add-In XLSTAT 2009.1.02 is provided as Excel-file (CSV). The data include file name, sample name, type of treatment, area, calculated N2O amounts and statistical values)

Figshare: Validation with Reference samples 2017. https://doi.org/10.6084/m9.figshare.8143016.v1 ^10^

This project contains the following underlying data:

- Validation with Reference samples 2017.csv (Raw data of 7 reference samples of 2017 of the "validation section" are provided as Excel-file (CSV). The data include file name, sample name, certified type of treatment and calculated N2O amounts (single and average values))

Figshare: Market survey 2019 rawdata. https://doi.org/10.6084/m9.figshare.8143031.v1 ^11^

This project contains the following underlying data:

- Market survey 2019 rawdata.csv (Raw data and descriptive statistic data of the "market survey section" performed with the Add-In XLSTAT 2009.1.02 are provided as Excel-file (CSV). The data include file name, sample name, area, calculated N2O amounts, test result and statistical values)

Data are available under the terms of the Creative Commons Zero “No rights reserved” data waiver (CC0 1.0 Public domain dedication).
