# Supplementary material for: Identification of nitrite treated tuna fish meat via the determination of nitrous oxide by head space-gas chromatography/mass spectrometry
Source: F1000Res. 2019 Aug 12;8:711. Originally published 2019 May 22. [Version 2] doi: 10.12688/f1000research.19304.2 (PMC6611128; doi:10.12688/f1000research.19304.2)
Supplement: Peer review history

# Peer review: Reviewer response for version 1

**DOI:** 10.5256/f1000research.21163.r49712

**Published:** 2019-07-03

**Authors:** Maurus Biedermann

**Affiliations:** 1 Official Food Control Authority of the Canton of Zurich (Kantonales Labor), Zürich, Switzerland

The authors developed a rapid method for the detection of a nitrite treatment of tuna fish meat. It is a straight forward static headspace GC-MS method and may be most probably combined with the determination of carbon dioxide, another agent to stabilize the red color of the meat. Brining experiments with ^15^N labeled sodium nitrite proved the reaction path. The subject is of current importance and I suggest the indexing after minor revision.

**Minor comments:**

- Page 3, “introduction”: Is there the possibility of combining the determination of CO and N _2_O, e.g. even within the same GC run? If so, please mention that option/possibility.
- Page 3, “Samples and sample preparation”: Please provide more details on the quantification, how was the calibration performed (one-point calibration, calibration curve, range of calibration…)?
- Page 4, “method development”, first and second paragraph: Within the second paragraph of the chapter it was concluded N _2_O to be the analyte in terms of robust chromatography. That explains the procedure of the two-step reduction described in the first paragraph. The GC temperature program was adjusted presumably based on the stronger retention of N _2_O. Please revise order of chapters and/or conclusions.
- Page 5, figure 3 text: Please mention the value of the untreated samples (below LOQ?) within the figure text. 
- Page 5, “Validation with reference samples”: Please specify “free of N _2_O”; according to the supplementary data the findings ranged between 13-31 ug/kg N _2_O.

I confirm that I have read this submission and believe that I have an appropriate level of expertise to confirm that it is of an acceptable scientific standard.

# Response

**Published:** 2019-08-07

**Authors:** Markus Niederer

**Affiliations:** State Laboratory Basel-City, Switzerland

We would like to thank the reviewer for his time and constructive feedback.

1. As requested, we now mention the possibility of combining the determination of CO and N2O in the introduction.
2. We fulfilled the request by adding the expression „one-point calibration“.
3. Regarding the remarks on „method development“, we do not understand the line of thought: N2O not NO is the target compound. Compared to CO, N2O elutes only at higher oven temperatures. NO being a reactive intermediate is not amenable for GC analysis. With the first section explaining the fundamental differences between the detection of a CO treatment and a nitrite treatment and the second section providing details on the latter, we see no reason in revising the text.
4. The text of fiqure 3 was not changed because the required data is already mentioned in the text of the corresponding chapter.
5. “Validation with reference samples”: “free of N _2_O” is specified as lower than 30 µg/kg.

# Peer review: Reviewer response for version 1

**DOI:** 10.5256/f1000research.21163.r49814

**Published:** 2019-06-25

**Authors:** Ulrich Busch

**Affiliations:** 1 The Bavarian State Office for Health and Food Safety (LGL), Erlangen, Germany

Addition of nitrite to tuna aiming at fresh reddish looking tuna has resulted in consumption of old tuna products with allergic reactions due to the formation of histamine. A systematic use of nitrite injections to rejuvenate tuna meat has been identified. The article "Identification of nitrite treated tuna fish meat via the determination of nitrous oxide by head space-gas chromatography/mass spectrometry" is well written, the method seems sufficiently valid to me. We do not know of an alternative method to prove nitrite treatment.

 We would be very pleased if the manuscript could be indexed, as there is no published adequate method to prove the falsification.

I confirm that I have read this submission and believe that I have an appropriate level of expertise to confirm that it is of an acceptable scientific standard.

# Response

**Published:** 2019-08-07

**Authors:** Markus Niederer

**Affiliations:** State Laboratory Basel-City, Switzerland

We would like to thank the reviewer for his time and his important comments.
